# Supplementary material for: Influence of heterogeneous age-group contact patterns on critical vaccination rates for herd immunity to SARS-CoV-2
Source: Sci Rep. 2022 Feb 16;12:2640. doi: 10.1038/s41598-022-06477-0 (PMC8850460; doi:10.1038/s41598-022-06477-0)
Supplement: Supplementary file 1 — Supplementary Information. [file 41598_2022_6477_MOESM1_ESM.pdf]

Supplementary information of "Influence of heterogeneous age-group contact patterns on critical vaccination rates for herd immunity to SARS-CoV-2" by J. Saldaña and C. Scoglio.

Tables

In Table S1, we report the contact data matrices used to derive our results. Contact data have been downloaded with the default settings, such as all contacts for day type, contact duration, contact intensity and all genders. Reciprocity, weigh by age, weigh by week/weekend, include supplemental professional contacts, and all locations have been checked.

In particular, reciprocity means that contacts are reciprocal and, hence, the total number of contacts per unit of time of individuals of age group i with individuals of age group j has to be equal to total the number of contacts per unit of time of individuals of age group j with individuals of age group i. This is equivalent to impose that $c_{ij} f_i = c_{ji} f_j$ where $C = (c_{kl})$ is the contact matrix and f_k is the fraction of the population in the age group k.

For each country, the population fraction f_k of each age group used in our analysis is shown in Table S2.

Country	Age group	contact $[0,18)$	contact $[18,60)$	contact 60+	total contacts
Belgium	$[0,18)$	5.426	5.143	0.772	11.341
	$[18,60)$	1.864	9.383	1.540	12.787
	60+	0.723	3.982	3.175	7.880
Germany	$[0,18)$	4.045	3.907	0.567	8.519
	$[18,60)$	1.219	6.216	0.998	8.433
	60+	0.408	2.299	2.127	4.834
Italy	$[0,18)$	12.753	8.990	1.513	23.256
	$[18,60)$	2.669	13.530	2.594	18.793
	60+	1.032	5.957	3.558	10.547
Netherlands	$[0,18)$	11.181	5.649	0.681	17.511
	$[18,60)$	2.108	10.714	1.491	14.313
	60+	0.775	4.548	3.464	8.787
Peru	$[0,18)$	13.855	6.664	0.792	21.311
	$[18,60)$	4.369	6.559	1.039	11.967
	60+	3.210	6.422	1.262	10.894
Zimbabwe	$[0,18)$	5.580	3.811	0.524	9.915
	$[18,60)$	4.025	6.617	0.825	11.467
	60+	5.861	8.737	2.007	16.605

Table S1. Daily contact matrices used in the numerical integration. The last column gives the sum of each row which is equal to the total per capita daily number of contacts.

Age group	Belgium	Germany	Italy	Netherlands	Peru	Zimbabwe
$[0,18)$	0.207	0.179	0.172	0.219	0.361	0.491
$[18,60)$	0.572	0.572	0.577	0.588	0.550	0.465
60+	0.221	0.249	0.251	0.193	0.089	0.044

Table S2. Population fraction of each age group for the considered countries.

Additional contour plots

To complement the contour plots presented in the main text, Figure S1 shows a choice of values of the parameters that increases the heterogeneity among the three age groups: lower waning rates of immunity for the age groups 1 and 3, and slightly different probabilities of successful protection of the vaccine for each age group. Such heterogeneity implies that the vaccination strategy that maximizes the vaccination coverage under the constraint $\bar{w} = \bar{w}_c$ (green points) is now clearly distinguishable from the uniformly random vaccination strategy (red points).

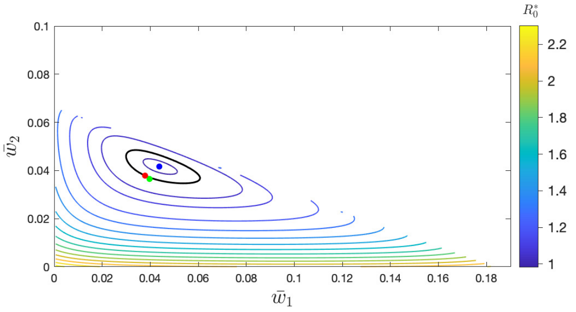

(a) Belgium

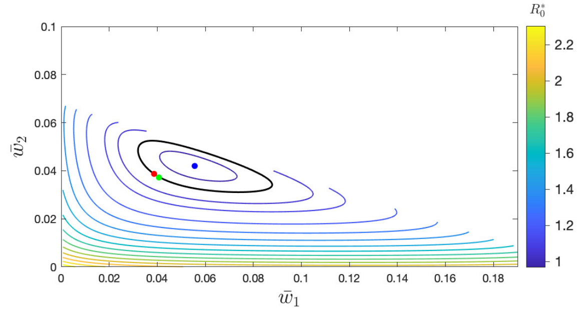

(b) Germany

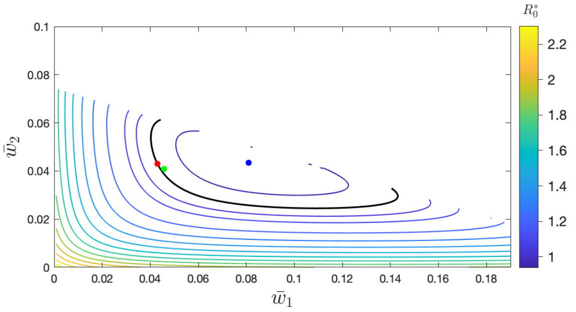

(c) Italy

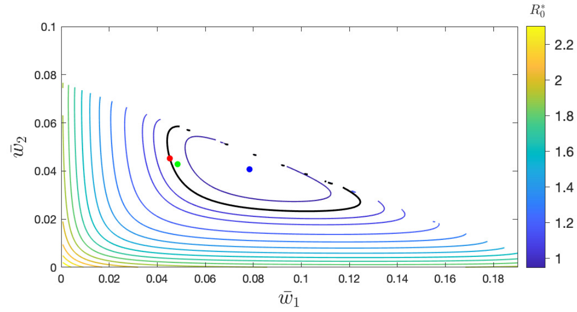

(d) Netherlands

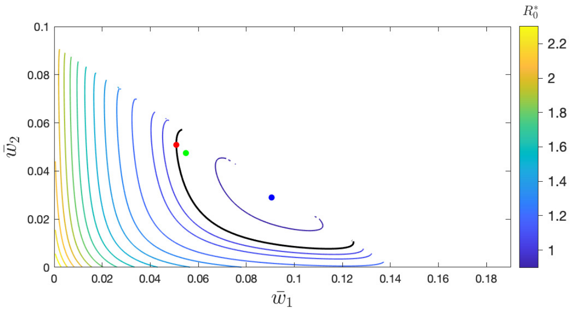

(e) Peru

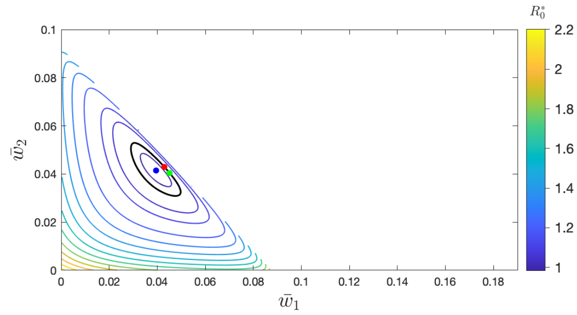

(f) Zimbabwe

Figure S1. Contour levels of $R_0^*(\bar{w}_1, \bar{w}_2)$ at the DFE of system (1) with $p_1 = 1$, $p_2 = 0.95$, and $p_3 = 0.9$. Black level curve corresponds to $R_0^* = 1$. Blue point: Minimum of R_0^*. Red point: R_0^* under uniformly random vaccination ($\bar{w}_1 = \bar{w}_2 = \bar{w}_c$). Green point: R_0^* at the disease-free equilibrium with the maximum vaccination coverage. Parameters: $\gamma_1 = 1$, $\gamma_2 = 1$, $\gamma_3 = 0.9$, $\delta_1^v = 1/26$, $\delta_2^v = 1/52$, $\delta_3^v = 1/26$. For each country, β is scaled such that $R_0 = 2.5$ in the absence of vaccinated individuals.
